# Structure, Function, and Genetics of the Cerebellum in Autism

**DOI:** 10.20900/jpbs.20220008

**Published:** 2022-10-25

**Authors:** Lindsey M. Sydnor, Kimberly A. Aldinger

**Affiliations:** 1 Center for Integrative Brain Research, Seattle Children’s Research Institute, Seattle, 98101, WA, USA; 2 Department of Pediatrics, Division of Genetic Medicine, University of Washington, Seattle, 98105, WA, USA

**Keywords:** autism, cerebellum, Purkinje cells, cytoarchitecture, excitatory inhibitory imbalance, genetics

## Abstract

Autism spectrum disorders are common neurodevelopmental disorders that are defined by core behavioral symptoms but have diverse genetic and environmental risk factors. Despite its etiological heterogeneity, several unifying theories of autism have been proposed, including a central role for cerebellar dysfunction. The cerebellum follows a protracted course of development that culminates in an exquisitely crafted brain structure containing over half of the neurons in the entire brain densely packed into a highly organized structure. Through its complex network of connections with cortical and subcortical brain regions, the cerebellum acts as a sensorimotor regulator and affects changes in executive and limbic processing. In this review, we summarize the structural, functional, and genetic contributions of the cerebellum to autism.

## INTRODUCTION

Autism spectrum disorders (ASD) are a group of phenotypically and genetically heterogeneous neurodevelopmental disorders that are diagnosed by core deficits in social communication and the presence of repetitive, stereotyped behaviors [1]. Individuals diagnosed with ASD exhibit a wide range of clinical features that vary in severity. Notably, motor impairment is the most common non-diagnostic clinical feature observed among individuals diagnosed with ASD [2–4].

Mounting evidence has implicated the cerebellum in ASD due its central role in regulating sensorimotor functions [5–7]. However, the cerebellum’s influence far exceeds this classically-defined purpose. Studies over the last few decades have unearthed the cerebellum’s essential responsibilities in motor and nonmotor learning, higher executive functions, affect regulation, language comprehension and production, social skill, visual-spatial performance, and memory functions [8–11]. Aberrations in cerebellar function have extensive overlap with ASD symptomology; both ASD and cerebellar dysfunction can result in adverse effects on language, visual-spatial performance, working memory, executive function, and affect regulation [1,2,7,10].

Autism is now diagnosed early in childhood, often prompted by delayed milestones [12]. Thus, atypical brain function begins long before neurons and their connections are fully mature. As such, essential questions in the field remain. ASD is a clinical diagnosis that relies on detecting the presence or absence of symptoms present in a child. In the absence of biomarkers for ASD testable prior to symptom onset (e.g., prenatally), it is not yet clear when neurodevelopmental processes first deviate from neurotypical development. Concurrently, the extreme diversity of ASD features and great overlap of symptomology with cerebral functions makes cerebello- vs cerebro-specific contributions to dysfunction important—but difficult—to disentangle.

Here we discuss how abnormalities in the cerebellum of individuals diagnosed with ASD may give rise to clinical features. We consider altered cytoarchitectural findings, subcortical and cortical projections, and gene expression patterns in the cerebellum of individuals with ASD. We examine how these findings interplay with the excitatory/inhibitory imbalance hypothesis of autism.

## CEREBELLAR DEVELOPMENT

**Figure 1.**
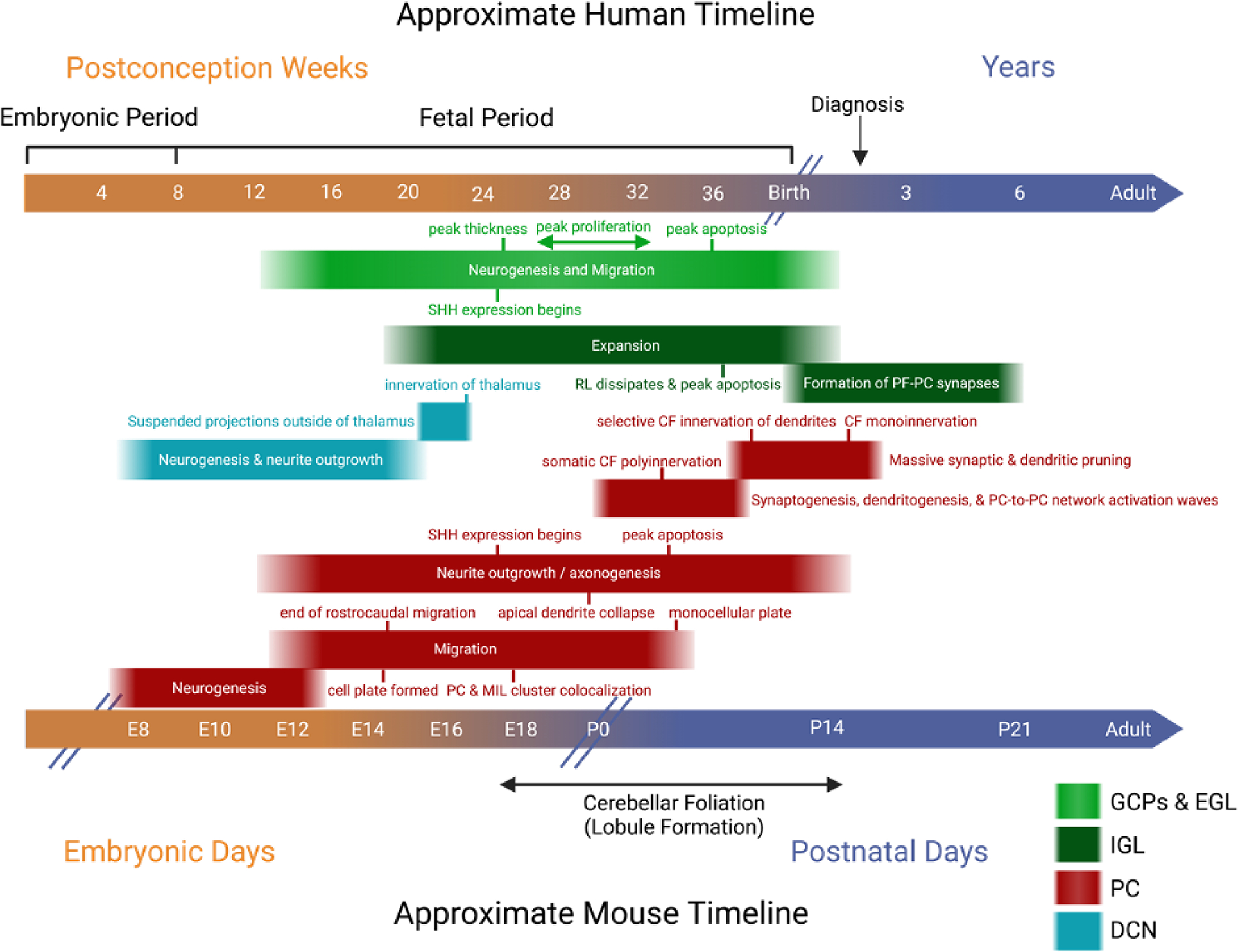
Approximate human and mouse timelines of relevant Purkinje cell (PC), deep cerebellar nuclei (DCN), and granule cell development. CF = Climbing Fiber; GCPs = Granule Cell Precursors; EGL = External Granular Layer; IGL = Internal Granular Layer; MLI = Molecular Layer Interneuron, RL = Rhombic Lip. Relationships between the timeline of developmental landmarks may be causative, in some cases. For example, coexpression levels of SHH (sonic hedgehog) in granule cells and PCs have been shown to regulate cerebellar foliation initiation and lobule number [21] and both proliferation of granule cell precursors and thickness of the PC layer [22]. An approximate third-trimester-to-birth timeline for the potential existence of human traveling PC-to-PC activation waves in the developing cerebellum is depicted, as activation waves among expansive monosynaptic PC-to-PC connection networks, although not explored in this paper, can be found up to 2 postnatal weeks in mice [23]. Figure inspired by Contractor et al. [24]. Other sources: [1,15–18,25–33].

In humans, cerebellar development begins in the embryonic period, continues through the fetal period, and completes its volumetric growth arc after about a decade of postnatal life [13,14]. Two progenitor niches are established sequentially: first, the ventricular zone and second, the rhombic lip [15]. These proliferative regions give rise to GABAergic and glutamatergic neurons, respectively. After neurogenesis in proliferative niches, cerebellar cortical neurons migrate radially and tangentially. GABAergic Purkinje cells (PCs) exiting the ventricular zone migrate along a scaffold of radial glial fibers towards the pial surface, while glutamatergic neurons, including granule cells, initially migrate tangentially; both pathways are mediated by distinct signaling molecules [16–18]. Glutamatergic granule cells later migrate radially inwards along Bergmann Glial fibers, the descendants of radial glia [19]. Each cell type follows a unique developmental timeline of neurogenesis, migration, neurite development, and synaptogenesis [13,15,20]. The details for each of these processes remain to be meticulously defined, as studies of fetal tissue are limited, and intact samples are especially difficult to acquire in the third trimester. However, timelines with respect to PCs and internal and external granule cells within the broader context of the developing cerebellum are approximated in Figure 1.

## CEREBELLAR STRUCTURE

**Figure 2.**
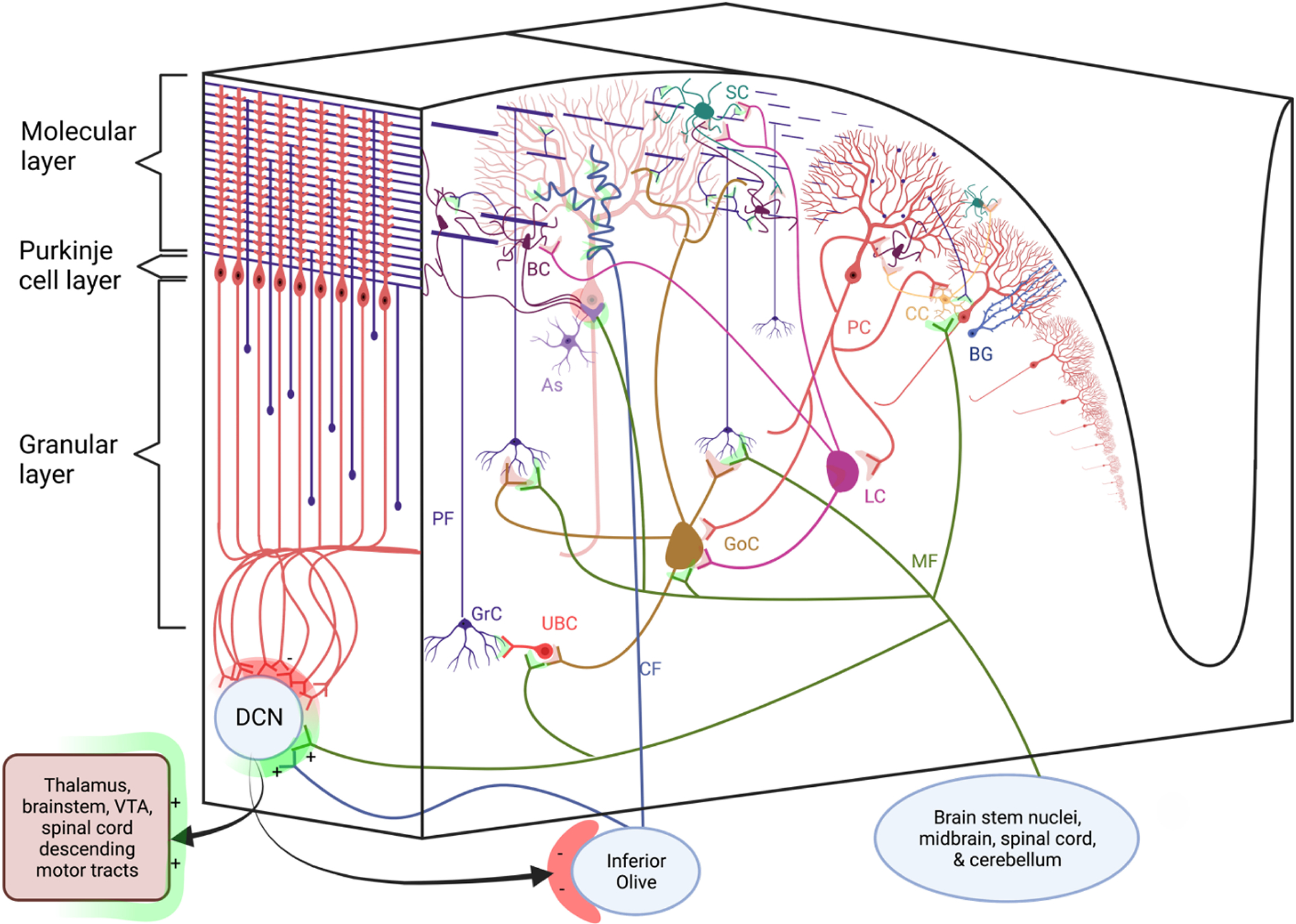
The cytoarchitecture and circuitry of the human cerebellar cortex. Purkinje cells (PCs) send one or two primary, fan-like dendrites into the molecular layer, extending in a plane orthogonal to the long axis of parallel fibers (PFs) [38]. PFs of granule cells (GrCs) form synapses with PCs. PCs also receive input from climbing fibers (CFs) that carry information from the brain stem nuclei, midbrain, spinal cord, and recursive inputs from the cerebellum. GrCs receive input from many cells, including cerebellar afferents called mossy fibers (MFs) from brain stem nuclei and spinal cord. The monolayer of PC somas, the Purkinje cell layer, also includes candelabrum cells (CCs) [39] and the somas of Bergmann glia (BG). PCs receive inhibitory inputs from stellate cell (SC) and basket cell (BC) molecular layer interneurons, at PC distal dendrites and somas, respectively. BC projections to PC somas are located within pinceaux formations involving astrocytes (As), depicted in purple. Pinceau are an active area of research that may be especially pertinent in the future of ASD studies and cerebellar development [40]. SCs form synapses at PC distal dendrites while BCs connect with the proximal dendrites and somas of PCs [35]. PCs integrate cerebellar cortical information and send their output to the deep cerebellar nuclei (DCN). DCN project to many cortical, subcortical, and descending motor and somatosensory targets. GrCs, unipolar brush cells (UBCs), globular cells (GCs), Golgi cells (GoCs), and Lugaro cells (LCs) are also found in the granular layer. Excitatory projections are marked with green at their synapses, whereas inhibitory projections are marked with red. Other sources: [41–43].

The resultant mature cerebellar circuit has a repeated, uniform cytoarchitecture. As shown in Figure 2, the cerebellum receives two distinct afferents: excitatory climbing fiber (CF) inputs from the inferior olive and excitatory mossy fiber (MF) inputs from the brain stem nuclei, midbrain, spinal cord, and recursive cerebellar connections. CF and MF inputs send collaterals directly to the deep cerebellar nuclei (DCN) and project into cerebellar cortical layers. MFs’ primary projections are to granule cells, whose axons bifurcate in the molecular layer into parallel fibers (PFs). Collectively, these PFs form hundreds of thousands of excitatory synapses with PCs [33], as each PF forms 1–2 synapses at the distal dendrites of each PC [34]. The only other cerebellar afferent, CFs, project directly to PCs, weaving around and innervating their soma and proximal smooth dendrites [35,36]. A single CF innervates each PC in neurotypical individuals, forming hundreds to thousands of synapses on the PC dendrite and soma [37].

**Figure 3.**
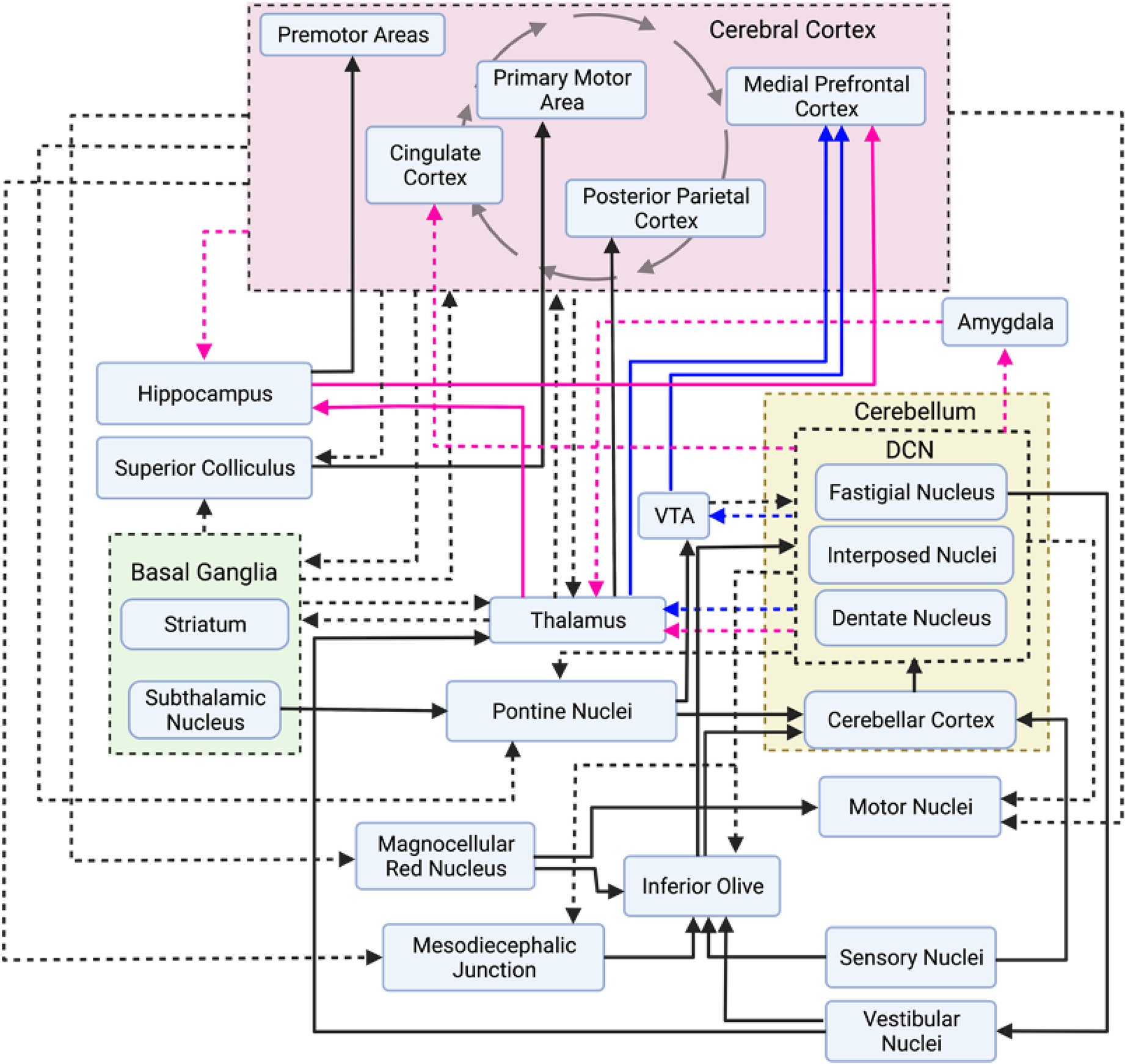
Simplified circuit diagram of cerebellar projection pathways. Efferent projections from a group of nuclei or a grouped region are represented with dashed arrows. Projections from non-grouped regions/nuclei are represented with solid arrows. Cerebellar projections to socially relevant areas of the brain are also outlined in pink and blue arrows. Pink arrows represent limbic system projections, and blue arrows represent projections to dopaminergic areas. Importantly, not all monosynaptic projections as outlined may be true monosynaptic projections. Further work will elucidate the true connectivity patterns of different brain regions. Sources: [42,47–50].

Molecular layer interneurons (MILs), which include basket and stellate cells, are the main inhibitors of PCs. PCs are the only output of the cerebellar cortex [44] and primarily serve to inhibit DCN. The DCN then project to the cortical and subcortical networks via mono- and polysynaptic circuitry with the majority of their primary connections at the thalamus, while simultaneously projecting to descending motor and somatosensory targets (see Figure 2 for more details) [45–50]. DCN output is largely, although not exclusively, excitatory [45]. Excitatory projection neurons, constituting 50–60% of the total DCN cell population, make polysynaptic cerebellocerebral projections through the thalamus and extend projections to other subcortical structures as shown in Figure 3 [46]. Inhibitory DCN projection neurons, comprising 30–35% of all DCN cells, project extensively to the inferior olive, creating a strong cerebellar feedback loop. Many of the remainder of DCN neurons are local inhibitory DCN interneurons.

## CEREBELLAR STRUCTURE AND CONNECTIVITY IN ASD

Unveiled in convergent evidence from diffusion tensor imaging, functional magnetic resonance imaging, and lesion-based symptom mapping, the cerebellum has many higher-order sensory, motor, cognitive, and limbic connections. The simplified circuit diagram depicted in Figure 3 shows some mono- and polysynaptic projections of the cerebellum to the cerebral cortex and to subcortical structures, including some reciprocal cerebrocerebellar connections. Although not an exhaustive diagram, these regions could be drastically and immediately affected by alterations in cerebellar output.

Both neuroimaging and histopathological studies have documented atypical structure and functional connectivity of the cerebellum that correlate findings with severity of ASD symptoms. Hypoplasia of the posterior cerebellar vermis with an enlarged cisterna magna was first described in a 21-year-old man diagnosed with autism without intellectual disability [51]. Subsequent human neuroimaging studies support both posterior vermis hypoplasia [52–54] and mega cisterna magna [55,56]. ASD have shown decreases in the posterior vermis across diverse demographics [52,57], and hypoplasia of the posterior vermis has been shown to strongly predict ASD evaluation scores [58]. Some of these abnormalities are less prevalent or disappear altogether at later ages, consistent with the notion that development is blunted at young ages in ASD. The variability noted across studies is likely also due to the genetic and observed phenotypic diversity among diagnosed individuals and the differing ages of study participants.

Further age-dependent trends in ASD can be found in lobular volumes in structural MRI studies, with findings often limited to certain younger age groups [59,60]. Broadly, studies investigating age-based trajectories have found overgrowth in early development followed by either degeneration or retention of abnormal connections in adolescence and adulthood. Studies of white matter integrity and atypical connective patterns in different age groups have detected aberrations both in the cerebral cortex and in the cerebellum of adolescents with ASD, suggesting that arrested development may occur in late childhood or early adolescence [5,61,62]. The connective abnormalities often present as reduced lateralization of typically asymmetrical processes and could be the result of abnormal retention of early-developmental connections or recruitment of extra computational power as compensation for developmental damage. Although too numerous to explore in detail here, a few examples of larger trends are noted below.

Additional functional connectivity studies have also found atypical lateralization patterns, including reduced lateralization to the left cerebral and right cerebellar hemispheres and vermes in ASD during language processing and production [61,62]. Although similarly bilateral in childhood, these language-based cerebellocerebral connections typically become very one-sided in the mature brain to an extent that is not found in ASD brains. Interestingly, in neurotypical children who suffer from a unilateral speech injury, this less-lateralized pattern correlates with the ability to recover language skills, acting as a beneficial adaptation [62]. In contrast, this reduced lateralization correlates with decreased language ability in ASD. Thus, it is considered maladaptive in this context.

Functional connectivity studies have detected noncanonical activity in cerebrocerebellar networks related to social interaction and language in adolescents with ASD. This is evident in multiple domains: global cerebellar increases in connectivity [63], increases in non-canonical cerebellocerebral and cerebrocerebellar connections between regions of differing domains (supramodal cerebral connections to classically sensorimotor cerebellar regions, for example), and decreases in resting state connectivity between right Crus I/II and multiple expected contralateral supramodal regions of the cerebral cortex, as well as the thalamus, in functional MRI studies [63]. Each increase in network activity was significantly correlated with increased ASD behaviors, although these changes in network activity often do not persist into adulthood.

Further increases in neurotypically out-of-network cerebellocortical functional activity have also been identified in ASD. Findings include overconnectivity between cerebellar cortex and ipsilateral cerebral regions in a manner that falls outside of topographical principles of cerebellar organization, as contralateral cerebellocerebral connections are much more common in neurotypical individuals [63,64]. This is reflected, for example, in asymmetries found in fractional anisotropy (FA) studies of the cerebellar peduncles, which are bundles of myelinated axons connecting the cerebellum to other brain regions. Specifically, asymmetries between the inferior and middle cerebellar peduncles have been found in individuals with ASD [5,65,66]. FA findings indicated different density, inflammation, or myelination of white matter than expected of neurotypical individuals between comparative peduncles (e.g., between left and right inferior cerebellar peduncles). Many other aberrant connections have also been identified in ASD, including additional connections of classically considered nonmotor areas of the cerebellum to sensorimotor cerebral cortices: particularly, regions of the occipital lobe, premotor and primary motor cortices, and primary somatosensory cortex [63]. Atypical eye gaze [67], delayed orienting [68], impairments in smooth pursuit [69], altered movement perception, and deficits in facial perception [70] demonstrated by individuals with ASD (details reviewed by [71,72]) are likely mediated by these abnormal sensorimotor connections, other alterations in olivofloccular circuitry [73], and altered PC activity and number. In rodents, some PC projections bypass the DCN and synapse directly within the vestibular nuclei, suggesting that PC alterations in ASD could directly exacerbate vestibuloocular reflex abnormalities [74,75]. Additional out-of-network connections have also been found in ASD, including strong, atypical connections between right anterior cerebellum (lobules IV/V), which would normally predominantly project to somatosensory networks, and the left middle frontal gyrus, which is normally associated with literacy [5]. Collectively, preliminary findings in functional connectivity studies of ASD indicate the need for age-based studies of these larger volumetric and connective changes.

## EXCITATORY/INHIBITORY IMBALANCE AND PURKINJE CELL CHANGES IN AUTISM

Higher excitation to inhibition (E/I) ratios have been extensively reported in the cerebral cortex of individuals with autism [76–78]. Robust evidence supports projection-based and neuronal-circuit-based alterations that could serve as the foundation for E/I imbalances. Alterations in glutamatergic and GABAergic signaling, which have been robustly demonstrated among orthogonal studies, result in higher E/I ratios in the cerebral cortex of those with autism and subcortical structures [79–83]. Although the cerebral cortex has historically been the focus of E/I imbalance studies, ASD-specific alterations in the cerebellum could have broad influence on the overall E/I imbalances found in the cerebral cortex of individuals with autism.

The cerebellum’s vast connective network to large-scale cortical and subcortical networks could serve to propagate massive excitatory imbalances throughout the brain and is vastly affected by alterations in PCs in autism. The role of the PC is to integrate multiple time-sensitive, complex inputs from throughout the cerebellar cortex, create appropriately-timed simple [84,85] and complex spikes [86,87], and serve as the only output of the cerebellar cortex [44]. Therefore, there is no capacity to multiplex cerebellar cortical output and thus no room for PC error without compromising the entire functionality of the cerebellum. Thus, cerebellocortical outputs are heavily affected in most individual with ASD due to well documented reductions in PC number and morphology, which are summarized below.

**Figure 4.**
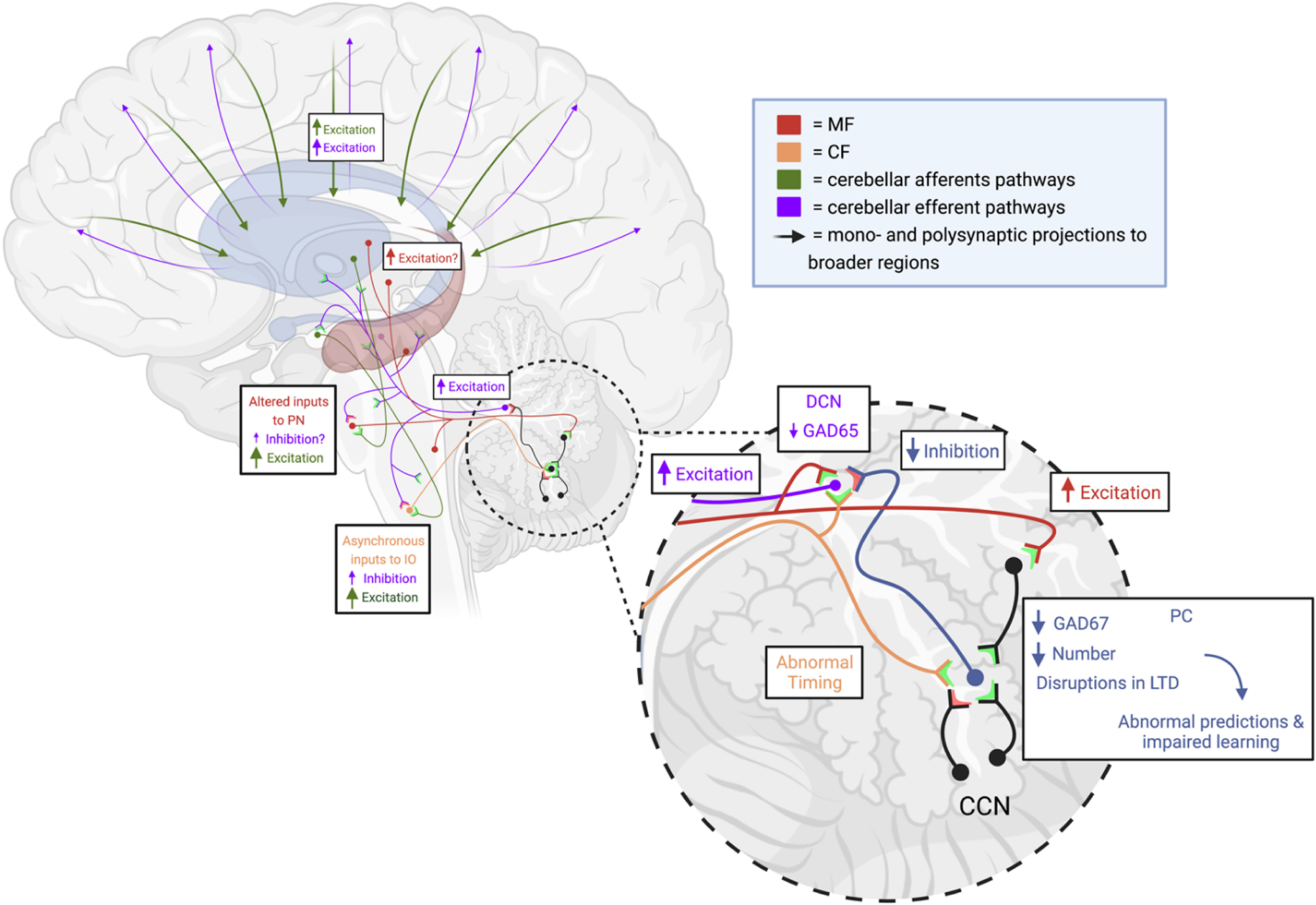
Proposed impact of cerebellar cortical GABAergic expression alterations on recursive cerebellar, cerebellocerebral, and cerebrocerebellar circuits. Reductions in deep cerebellar nuclei (DCN) inhibition, due to reduced Purkinje cell (PC) output may result in increased excitation of downstream excitatory targets of the DCN. These targets also serve to reciprocally excite the cerebellar cortex through mossy fiber (MF) connections, further altering the cerebellocortical computational relationship. Reductions in PC inhibition may alter inferior olive (IO) neurons’ rhythmicity by reducing activity in IO climbing fiber (CF) outputs. However, reductions in intrinsic dentate nuclei expression of GAD65 may decrease inhibition to IO neurons [96]. Subbranches are pathway representations, not eminences from singular neurons. Arrow size is a representation of relative presumed impact. CCN = cerebellar cortex neurons of types other than those explicitly discussed. Figure adapted from Mapelli et al. [8].

Convergent lines of evidence have repeatedly documented alterations in PCs in ASD. Studies of human postmortem cerebellum from individuals with ASD detected reductions in PC size, density, and expression of the GABAergic precursor, glutamate decarboxylase 65 (GAD65) and GAD67 [8,76,88,89]. At the same time, increased GAD67 transcripts have been noted in cerebellar interneurons [90]. Reductions in GAD65 and GAD67 transcripts and GABA receptor densities have been detected in the cerebral cortex and subcortical areas of postmortem ASD tissue [76,91]. GAD65 is a predominantly dormant apoenzyme that is important for fast modulation of inhibitory transmission during intense bouts of activity, whereas GAD67 is responsible for the majority of GABA synthesis [92]. Thus, as a fast-spiking inhibitory neuron [93], reduced GAD65 and GAD67 production severely limit proper PC function. In mice lacking either GAD67 or GAD65, PCs and basket cell interneurons are correlated with higher paired-pulse ratios of inhibitory post-synaptic currents, lower decay time constants, and deterioration of intracortical cerebellar basket cell to PC connections [92]. Additionally, altered GAD expression levels are known to impact synaptic plasticity throughout the cerebellar cortex [94]. Therefore, reductions in GAD are of massive concern for broad cerebellar computation and function and they may drastically affect the DCN and its downstream targets (Figure 4). Compensatory mechanisms may exist in human cerebellar efferent pathway as in mouse models [95] but remain to be investigated.

These PC-specific alterations are coupled with other known cerebellocortical neuronal circuit abnormalities that may further exacerbate E/I imbalances and ASD symptomology. This includes the retention of, on average, four CF inputs per PC whereas neurotypical cerebella retain only a single CF afferent per PC [35]. In neurotypical development, granule cell input contributes to the pruning of supernumerary CF inputs in a process beginning during the second trimester and continuing up until two postnatal years [43]. This CF retention in ASD has many potential implications on cerebellar function, as each CF has hundreds of synapses with each PC in neurotypical individuals, and CFs are responsible for inciting the largest depolarizing response of any neuron in the human body: the PC complex spike [97,98]. Thus, this retention of multiple CF inputs may irreparably alter PC computations, contributing to cerebellum-wide dysfunction.

PC afferents may be further altered in ASD. Their loss likely occurs after the development of cerebellar MLIs in ASD, as proper migration of basket and stellate cells rely upon PC signaling, and no cytoarchitectural MLI abnormalities—density of MLIs or proportion of MLI to PCs—have been found based on sampling right Crus II in ASD [89]. However, more studies on MLIs are required to verify these results and to expand our knowledge of regional alterations in MLI density and MLI expression patterns.

Ultimately, this reduction in GABAergic transmission and reports of glutamatergic alterations [76,99] (reviewed in [81]), not explored here, may predispose the developing cerebellum to larger network E/I balances, as the formation and refinement of synaptic inputs is dependent upon GABAergic activity during development [92]. Thus, abnormal cytoarchitecture can arise from large-scale alterations in GABAergic activity in the cerebellar cortex and other brain areas during the prenatal period.

## GENETIC CONTEXT OF THE CEREBELLUM IN ASD

ASD is highly heritable [100]. This feature has led to numerous studies that together define the genetic architecture of autism (reviewed in [101]). ASD displays considerable heterogeneity with both common and rare genetic variation in hundreds of genes likely contributing to the clinical variability observed among diagnosed individuals. Despite this complexity, pathway enrichment among ASD-implicated genes suggests some functional commonalities, including impairments in synapse function and chromatin modification [102]. Even so, these important molecular clues into the etiology of ASD provide limited information regarding the time periods, brain regions, cell types, and circuits that are likely to be impacted by these genetic events.

Gene expression studies of the developing human brain provide an important resource for preliminary investigation of where and when ASD-implicated genes are likely to act. Enrichment of high-confidence ASD gene expression has repeatedly detected peak expression in prenatal development and implicated excitatory pyramidal neurons and inhibitory interneurons in the neocortex and medium spiny neurons of the striatum [102–107]. Far fewer ASD gene enrichment analyses have implicated the cerebellum [103,106]. However, the cerebellum is not well represented in transcriptomic studies of the developing brain, including the commonly used BrainSpan dataset [108], which limits the statistical power to detect associations with the cerebellum.

**Figure 5.**
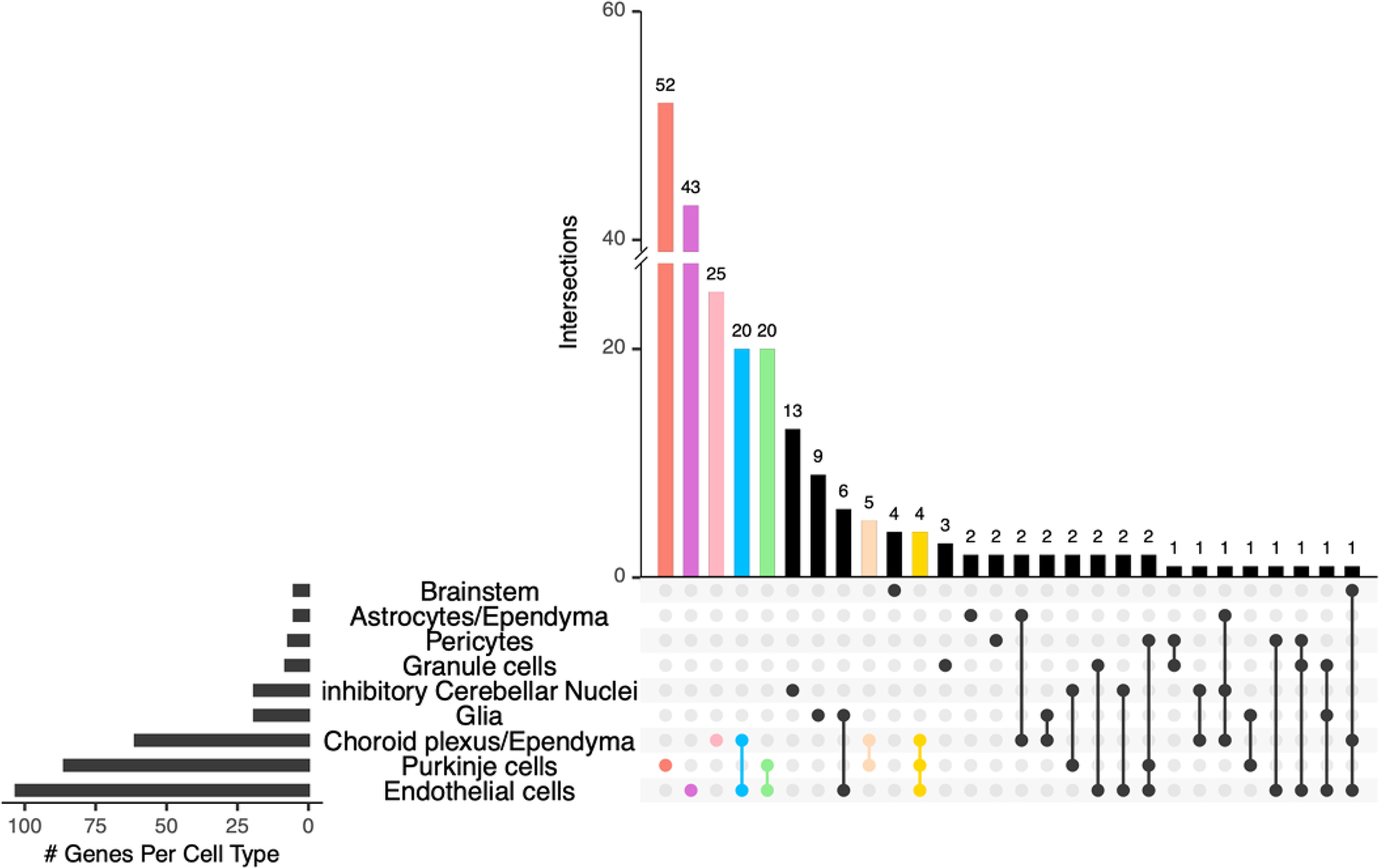
Distribution of ASD gene expression among cell types of the prenatal human cerebellum. To identify the cell type specificity of ASD gene expression, we compared the expression of each gene in a cell type relative to its expression in all other cell types using Seurat v4 [118] and the prenatal human cerebellum dataset [109]. This analysis revealed that 238 of the 345 ASD genes were significantly expressed in distinct cell types (average logFC >1.5 and FDR < 0.05). Overlapping expression of ASD genes among the top 9 cell types of the prenatal cerebellum is indicated by the numbers of genes (x-axis) and the numbers of genes per cell type (y-axis; circles with connecting lines indicate genes expressed in additional cell types); cell types with <5 genes expressed were omitted from the display. Colors correspond to cell type expression for the genes included in the protein-protein network, as shown in Figure 6.

**Figure 6.**
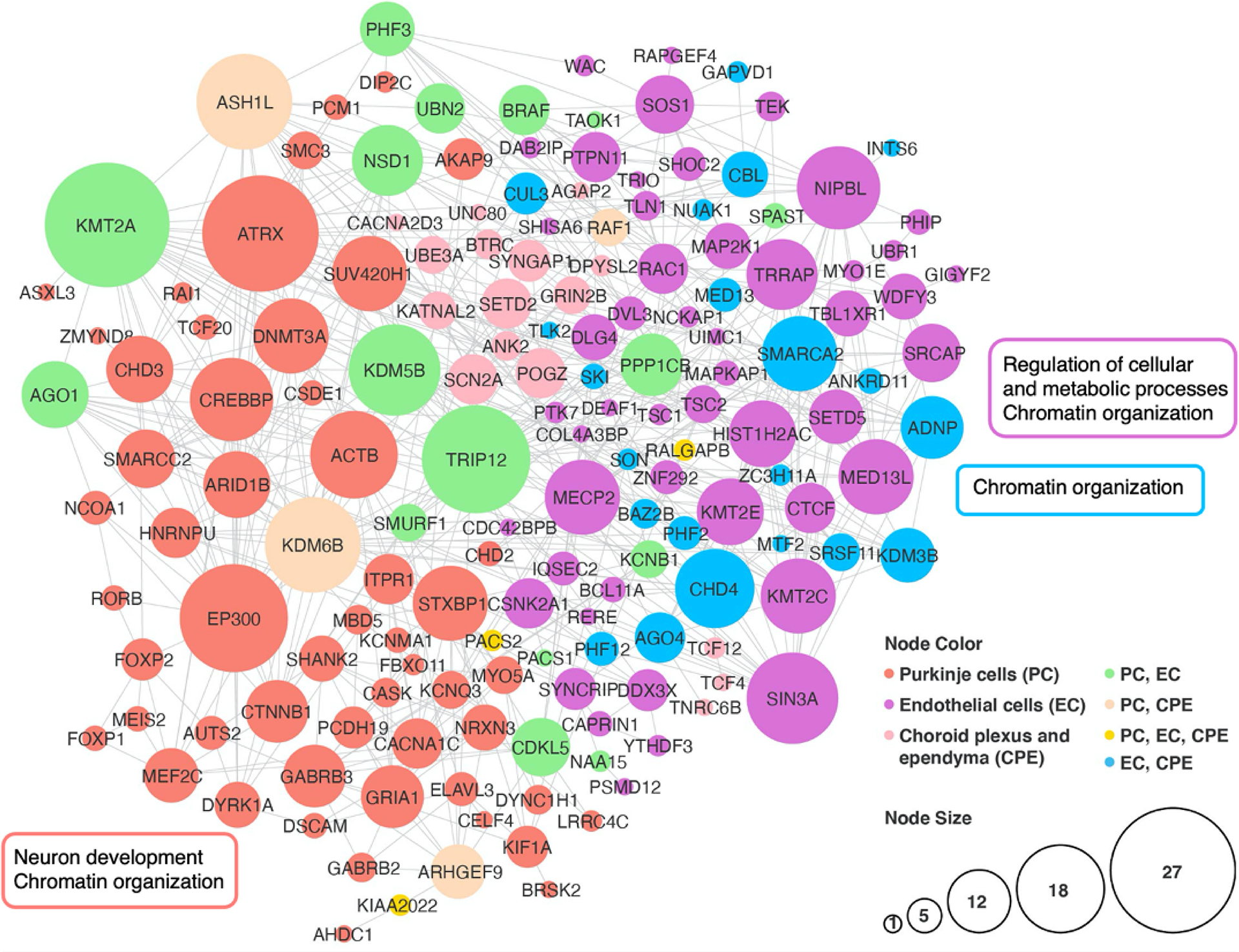
STRING protein-protein interaction analysis for three cell types of the developing cerebellum that express the most ASD genes. Genes are represented as nodes that are scaled in size relative to the number of interactions and colored based on cell type expression. Three STRING v11.5 [124] networks were constructed using the lists of ASD genes expressed in PCs, endothelial cells, and choroid plexus/ependyma then merged into a new network, retaining only the connected gene nodes. Functional annotation for gene sets is shown in rectangles; several gene sets did not have functional enrichment. Cytoscape v3.9.1 was used for network visualization [125].

New studies of human cerebellar development that apply single-cell technology at unprecedented scale provide an opportunity to examine the expression profiles of high-confidence ASD genes in the context of human cerebellar development. Investigation of 108 high-confidence ASD genes in a single-nucleus RNA-seq dataset of 69,174 nuclei from the prenatal human cerebellum spanning 9 to 21 postconceptional weeks detected prominent expression in neuronal and non-neuronal cell types, including PCs [109]. To extend this analysis, we compiled a list of 345 ASD genes from exome and genome sequencing studies [105,109–114] and the SPARK gene list [115] (Supplementary Table S1), then examined gene expression in the same human cerebellar development dataset (Figure 5). Among the 21 distinct cell clusters defined in the cerebellum dataset, ASD genes were significantly expressed in 17 (81%) of them, with most ASD genes expressed in just three clusters: endothelial cells, PCs, and choroid plexus/ependyma. Consistent with previous studies, the subset of ASD genes that are expressed among these cell types in the cerebellum show enrichment of protein-protein interactions that converge on chromatin organization (Figure 6). Additionally, the protein network associated with ASD gene expression in PCs is enriched for neuron development, while the protein network in endothelial cells is enriched for the regulation of cellular and metabolic processes.

Initial ASD exome sequencing studies identified de novo rare variants in many genes, but only a few individuals accounted for the variants in any gene. Similarly, exome sequencing of individuals with cerebellar malformations identified de novo rare variants in many genes that were mostly unique to a single family [116]. The cerebellar malformation genes were associated with nearly all previously known genetic disorders, though a cerebellar phenotype had not been described for many of these syndromes. To more broadly investigate whether ASD genes have a known association with cerebellar impairment, we queried OMIM [117] and curated the clinical evidence for cerebellar malformations and cerebellar dysfunction. Of the 345 ASD genes queried, 219 (63%) were associated with a genetic disorder, and 99 (45%) reported at least one patient with abnormal cerebellar neuroimaging or ataxia. A cerebellar malformation was noted for 73 genetic disorders, mostly cerebellar hypoplasia and cerebellar atrophy; other malformations included Chiari I, Dandy-Walker malformation, and cerebellar cortical dysplasia. Ataxia was reported for 32 genetic disorders. The ASD genes associated with cerebellar malformations were highly expressed in PCs and endothelial cells, while the ataxia-associated genes showed less discrete expression. For example, *BCL11A* and *FOXP1* are expressed in a subset of PCs and endothelial cells [116], while *MBD5* and *TRIO* are moderately expressed in all 21 cell types of the developing cerebellum. Expanding the spatiotemporal analysis of these genes across cerebellar development is needed.

Cerebellar hypoplasia is often a nonspecific feature with variable penetrance that is associated with numerous genetically defined neurodevelopmental disorders [119]. However, deeper investigation of specific genetic disorders has revealed that cerebellar hypoplasia is a prominent feature in some of these disorders, including *BCL11A* and *FOXP1*, which are also notable ASD genes [116,120]. BCL11A and FOXP1 are transcription factors that are expressed in both the hematopoietic and central nervous systems, where they regulate progenitor cell proliferation, differentiation, and migration (reviewed in [121–123]). Their function and target genes have not yet been studied in the cerebellum, but *BCL11A* and *FOXP1* are highly expressed in the striatum, further complicating their impacts to motor and behavioral functions. Motor coordination deficits that are frequently documented in ASD may simply be denoted as ataxia in genetic disorders and are not captured consistently in focused single-gene studies. Thus, the extent to which cerebellar dysfunction contributes to the impact and prognosis of ASD is not well known and warrants further study among individuals with ASD and in phenotypic studies of ASD single-gene disorders.

## CONCLUSIONS

Convergent evidence across multiple studies and modalities demonstrates structural and functional impact to the cerebellum in ASD. Precise mapping between macroscopic and microscopic differences in the cerebellum with clinical features and subdomains of ASD is still emerging. Transcriptomic, histological, and functional studies converge on Purkinje cell dysfunction in ASD, but many of the other neuronal and non-neuronal cell types of the cerebellum have not been studied in ASD and require investigation. ASD-specific impairments in cerebellar function are reciprocally connected to impaired cerebral cortex function. Thus, the next frontier requires coordinating studies to disentangle the complex interplay between the cerebrum and the cerebellum in ASD.

## Supplementary Material

Supplementary Table S1

## Data Availability

The single-cell dataset analyzed in the study can be found at https://cbl-dev.cells.ucsc.edu [109] The gene list generated from the study is available in the Supplementary Table S1.
